# SARS-CoV-2 Detection Using Optical Fiber Based Sensor Method

**DOI:** 10.3390/s22030751

**Published:** 2022-01-19

**Authors:** Muhammad Usman Hadi, Menal Khurshid

**Affiliations:** 1 School of Engineering, Ulster University, Newtownabbey BT37 0QB, UK; 2 Akbar Niazi Teaching Hospital (ANTH), Islamabad Medical and Dental College (IMDC), Bharakahu, Islamabad 45400, Pakistan; menal.15@iideas.edu.pk

**Keywords:** SARS-CoV-2, COVID-19, optical fiber sensor, U-shaped probe, PCR, COVID-19 detection

## Abstract

The SARS-CoV-2 Coronavirus disease, also known as the COVID-19 pandemic, has engendered the biggest challenge to human life for the last two years. With a rapid increase in the spread of the Omicron variant across the world, and to contain the spread of COVID-19 in general, it is crucial to rapidly identify this viral infection with minimal logistics. To achieve this, a novel plastic optical fiber (POF) U-shaped probe sensing method is presented for accurate detection of SARS-CoV-2, commonly known as the COVID-19 virus, which has the capability to detect new variants such as Omicron. The sample under test can be taken from oropharyngeal or nasopharyngeal via specific POF U-shaped probe with one end that is fed with a laser source while the other end is connected to a photodetector to receive the response and postprocess for decision-making. The study includes detection comparison with two types of POF with diameters of 200 and 500 µm. Results show that detection is better when a smaller-diameter POF is used. It is also seen that the proposed test bed and its envisaged prototype can detect the COVID-19 variants within 15 min of the test. The proposed approach will make the clinical diagnosis faster, cheaper and applicable to patients in remote areas where there are no hospitals or clinical laboratories due to poverty, geographic obstacles, or other factors.

## 1. Introduction

Since the early 2020, world has faced tremendous challenges due to SARS-CoV-2 Coronavirus disease, also known as COVID-19 * (* Official names announced for the virus responsible for COVID-19 (previously known as “2019 novel coronavirus”). It is a contagious disease that has caused a novel strain of flu virus. The World Health Organization (WHO) reports that the first case of COVID-19 was first witnessed in Wuhan, China in late December 2019 [1]. By early February of 2020, all parts of the world were affected, and strict restrictions were in place. WHO had declared COVID-19 as a pandemic and it has been recognized as the biggest challenge in the 21st century [2].

The official name for the disease is coronavirus disease (COVID-19), and the virus, severe acute respiratory syndrome coronavirus 2 (SARS-CoV-2), attacks the immune system and infects the main respiratory tract of the human body. There is evidence that COVID-19 patients suffer from one of the following symptoms: coughing, fever, loss of taste and smell, tiredness, fatigue, diarrhea, and flu [3]. In fact, difficulty in breathing and loss of oxygen saturation are the most significant issues that COVID-19 patients suffer from day 3 to 8. Similarly, COVID-19 patients have seen involvement of kidney and heart failures as well [4]. Millions of people have lost their lives worldwide due to the inefficient and time-consuming process of diagnostic tests. The world has entered a stage in which the realization is present in society that we have to live with this virus and the only way is to take certain precautions in order to protect yourself from these issues.

In order to fight against this deadly virus, major pharmaceuticals have gained early success in development of COVID-19 vaccines and many countries have already vaccinated their citizens. It has been observed that the vaccination does not provide a foolproof defense from COVID-19 virus, however; it only makes the immune system strong enough to fight against the virus. 

Vaccination research is an ongoing process, and it will take some time to create a broad-spectrum vaccine that can counterfeit all the variants of COVID-19. However, the detection of infection is a high priority in the battle to conquer this pandemic. We are not only fighting against this deadly virus but also against time. The detection of COVID-19 through gene sequencing is possible using a technique called Reverse Transcription Polymerase Chain Reaction (RT-PCR) [5,6]. This procedure takes around 8 to 12 h (at priority), which leaves the patient anxious and in ambiguity since it is not clear if the patient has been infected or not. Additionally, the detection accuracy with PCR in first 3 days of getting exposed to COVID-19 is minimal [7,8]. The studies indicate that oropharyngeal swabs and sputum could improve the early detection of COVID-19 patients. Nevertheless, handling large amount of population, cost and equipment are the major limitations of this method. 

The early detection of COVID-19 will indeed improve the possibility of life expectancy of the COVID-19 patient, helping practitioners to make timely decisions and plan in a better way. Based on this, the use of optical technologies and sensors for early detection of COVID-19 has been the prime research area of investigation. Many different methodologies have been employed. 

The utilization of different methodologies to detect COVID-19 has become popular over the last year and a half. Examining the recent literature from Web of Science, we used the phrase “detection”, “diagnostic”, “optical”, “fiber” and “fibre” as searchable items. We obtained 21,845 items, out of which only three articles discussed the utilization of fiber-optic biosensors for COVID-19 detection. These articles proposed combination of an immunoassay with the plasmons and fluorescence detection of recombinant SARS-CoV protein N. It was shown that these technologies can lead to a greater sensitivity than the conventional method.

There are tests that can be used to see if someone has been infected with SARS-CoV-2: detection of viral nucleic acid and antigen (acute infection) and detection of antiviral antibodies (pre-infection). Even though there is a global debate about the use and misuse of antibody-detection tests, new tests with high sensitivity, specificity and low-cost technology are needed. As a result, postulating the invention of devices based on optical fibers is appropriate, and they may help manage this and upcoming pandemics with fewer negative consequences for society.

The general structure of a biosensor is made up of three distinct components: the substrate, biolayer and immobilization interface. The optical biosensor is a transducer in which the specimen under test interacts with the light that is passed or interacts with an optical fiber to produce a modulated optical signal with information related to the parameter being measured, which means fiber interacts with an external parameter and carries the modulated light signal from the source to the detector. From this modulated optical signal, the input measurement data may be retrieved. A wide range of immobilization techniques have been developed and employed for biosensing applications in recent decades. 

Fiber Bragg Gratings (FBG) [9], Long-Period Fiber Gratings (LPFG) [10], Surface Plasmon Resonance (SPR) [11], Lossy Mode Resonance (LMR) [12] and a broad range of interferometers [13] were shown to be viable for use as biosensors, with tapered fibers [14] and multimode fibers [13].

To the best of the authors’ knowledge, a novel plastic optical fiber sensor U-shaped probe-sensing method is presented and envisaged for detection of SARS-CoV-2 virus, and has the capability to detect new variants such as Omicron. The novelties of this article are as follows:

1. The U-shaped fiber-optic probe is realized from plastic optical fiber (POF) for two different diameters: 200 and 400 microns, respectively.
2. The test specimens are suggested to be taken from both oropharyngeal and nasopharyngeal tracts, as a sensing approach based on intensity modulation appears to be very cost effective with minimal logistics.
3. The envisaged prototype with its component analysis is shown, making it clear that the design of this prototype is simple and does not require any special training.
4. The results are shown in terms of intensity and relative intensity followed by comparison of the COVID-19 positive specimen with the proposed method and conventional PCR test method.
5. A validation of the method proposed is shown that confirms the accuracy of the proposed technique. In order to validate the proposed technique, it is important to verify that the proposed technique detects SARS-CoV-2 and not something random. In this research, the sample consisted of COVID-19 patients who presented to the hospital with respiratory symptoms.
6. A statistical analysis for the gathered data is presented to understand the cycle or map that is followed for COVID-19 detection in the hospital compared to the cycle of actions required in proposed detection.

Moreover, Section 2 presents in detail the emergence and features of COVID-19 that make it easier to understand the life cycle of COVID-19. Section 3 summarizes the main work employed for detection of COVID-19. Section 4 presents the U-shaped fiber-optic sensor methodology while Section 5 explains the Experimental testbed utilized for the work, followed by Section 6 highlighting the theoretical understanding behind the wavelength-bending radius and wavelength refractive index relationship. Section 7 presents the results while Section 8 presents the validation technique where comparisons of proposed and other techniques are compared. Section 9 compares the statistical analysis of the samples. Section 10 explains the future research direction and Section 11 concludes the paper. 

## 2. Emergence and Features of COVID-19

Coronaviruses are a diverse group of viruses that causes severe respiratory illnesses. In 2012, two types of zoonotic coronaviral high pathogens, SARS and MERS viruses, created a deadly respiratory sickness in humans and formed a new public health hazard in the twenty-first century. The public health community has been concerned about the evolution of coronaviruses [15,16,17,18]. Several health institutions in Wuhan, Hubei Province, China, observed clusters of individuals with pneumonia in late December 2019 [19]. Alpha coronavirus, Beta coronavirus, Gamma coronavirus and Delta coronavirus are the four genera of coronaviruses [18]. Recently, in November 2021, a new variant named the B.1.1.529 variant, commonly known as the Omicron variant, was reported in South Africa. Barely a month has elapsed since scientists in Botswana and South Africa alerted the world to a fast-spreading SARS-CoV-2 variant now known as Omicron. Researchers worldwide are racing to understand the threat that the variant—now confirmed in more than 20 countries—poses to the world. Yet it might take scientists weeks to paint a more complete picture of Omicron, and to gain an understanding of its transmissibility and severity, as well as its potential to evade vaccines and cause reinfections. Although genome sequencing is needed to confirm Omicron cases, some PCR tests can pick up a hallmark of the variant that distinguishes it from Delta variant. The emergence of Omicron has presented new challenges in the fight against SARS-CoV-2. A large number of mutations in the Spike protein suggest that its susceptibility to immune protection elicited by the existing COVID-19 infection and vaccines may be altered.

Coronavirus is an enveloped, RNA virus. Microscopically, prominent club-shaped spikes appear from its surface which form a halo (Corona) around the virus. Once inside the human respiratory tract, these spikes enable the virus to enter the cells for replication. The transmission of coronavirus mainly occurs via respiratory aerosols. The virus is acquired either by inhalation of the aerosols or direct contact with the eyes, nose or mouth. The incubation period for COVID-19, which is the time between exposure to the virus (becoming infected) and symptom onset, is on average 2–10 days; however, it can be up to 14 days. During this period, also known as the “presymptomatic” period, some infected persons can be contagious. Therefore, transmission from a pre-symptomatic case can occur before symptom onset. Virus adsorbs onto the cell membrane via surface spikes. The Angiotensin Converting Enzyme-2 acts as a receptor for the virus in the lower respiratory tract. The virus typically limits itself to the mucosal cells of the respiratory tract. The virus then enters the cell after losing its envelope and replicates in the cell cytoplasm. After replicating, the virus acquires its envelope from the endoplasmic reticulum of the cell. Once the replication is complete, the virus exits the cell and effects other cells. The angiotensin converting enzyme-2 is a crucial component of the renin–angiotensin–aldosterone system of the body, which is responsible for the regulation of the volume of bodily fluids. Binding of Coronavirus to the ACE-2 dysregulates the body fluid balance which causes diffuse edema of the respiratory tract, resulting in hypoxia. Involvement of the upper respiratory tract is characterized by Coryza (rhinorrhea), scratchy throat and low-grade fever. Involvement of the lower respiratory tract results in pneumonia, clinically manifesting as severe respiratory distress, fever, nonproductive cough, dyspnea and hypoxia.

**Figure 1:**
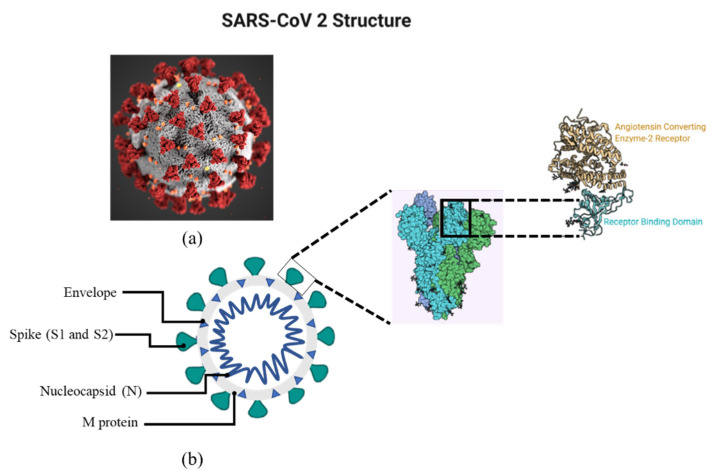
(**a**) shows the microscopic image of SARS-CoV-2 while (**b**) shows the structural diagram of SARS-CoV2. Structural schema of SARS-CoV-2 showing Spikes, envelope, nucleocapsid and M protein. The inset shows the structure of spike.

A spike (S), a membrane (M), an envelope (E), and a nucleocapsid (N) are the four different components of the virus (N). Among all reported RNA viruses, it possesses the biggest genome (26.4–31.7 kb). Single-stranded RNA is also found in the virus’ genetic material [20,21,22]. The structure of COVID-19 is shown in Figure 1.

**Figure 2:**
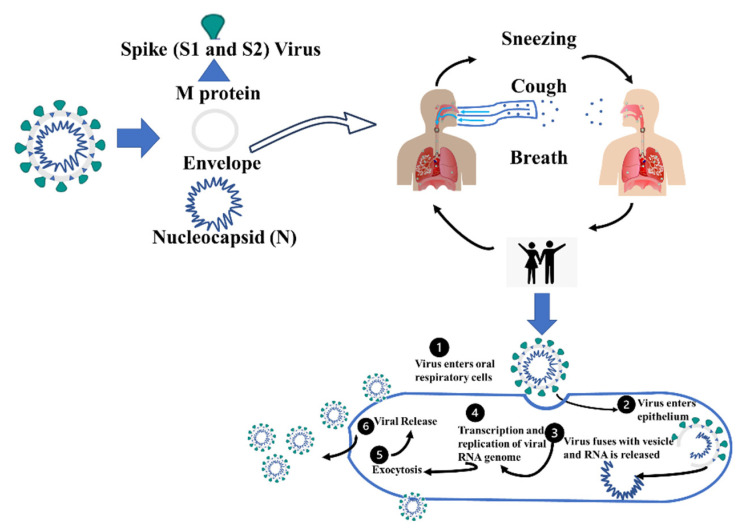
The infection cycle of SARS-CoV-2 (COVID-19). The life cycle summarizes the steps of how Coronavirus is transmitted and replicated.

The lifecycle of COVID-19 is summarized below in Figure 2. Generally, it spreads from the host through agents such as physical contact, sneezing, coughing and breathing droplets. These droplets can be transmitted to the respiratory tract (directly or through touch of the hand to the nose, eye and mouth area). 

## 3. Literature Review

In the following section, a discussion is included which summarizes the diagnostic techniques that have been proposed in the recent past (2020–2021). The methods/techniques are discussed in terms of type, details, efficiency, advantages and disadvantages in Table 1. 

**Figure 3:**
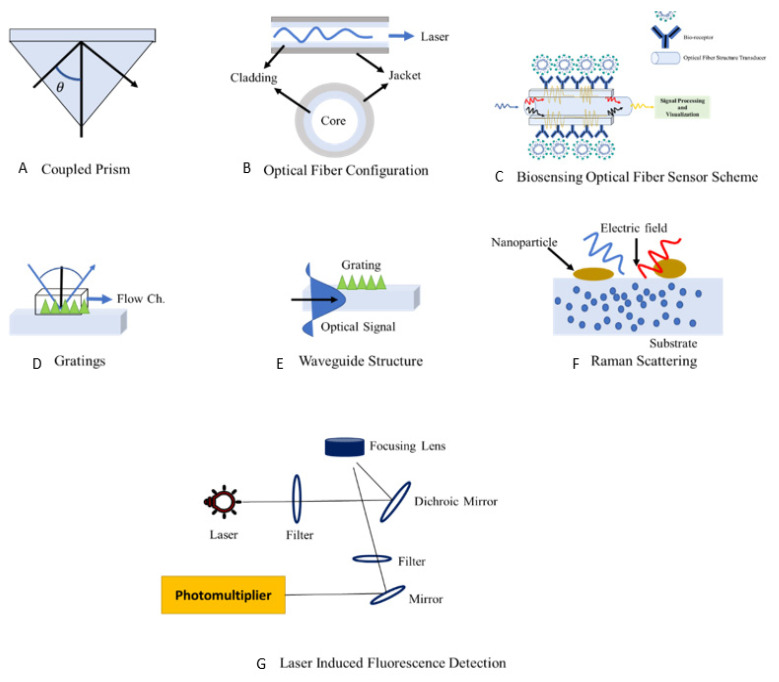
Block diagram of latest COVID-19 detection technologies structures: surface plasmon resonance (SPR) configuration (**A–D**); (**E**) bioreceptor fiber optic sensing method; (**F**) surface enhancement Raman scattering, and (**G**) laser-induced fluorescence (LIF) detector.

Figure 3 shows the methodologies employed recently that depict surface plasmon resonance (SPR) configuration (Part I to IV) such as traditional plasmonic based on a coupled prism, plasmonic based on grating (long/short) period and plasmonic based on a waveguide; Part V illustrates a fiber-sensing optical method using a biosensor with nanoparticles. Part VI illustrates surface-enhanced Raman scattering, employing nonpractical materials such as gold and graphene to boost viral sensitivity. The reflecting index (RI) and the wavelength of the laser have a nonlinear connection. Part VII displays structures of laser-induced fluorescence. As a result, altering the refractive index can lower the laser’s velocity wavelength in the optic structure. It is also crucial to control the amount of light reflected at the contact, as well as the critical angle for total internal reflection.

## 4. Fiber-Optic Sensing for COVID-19

**Figure 4:**
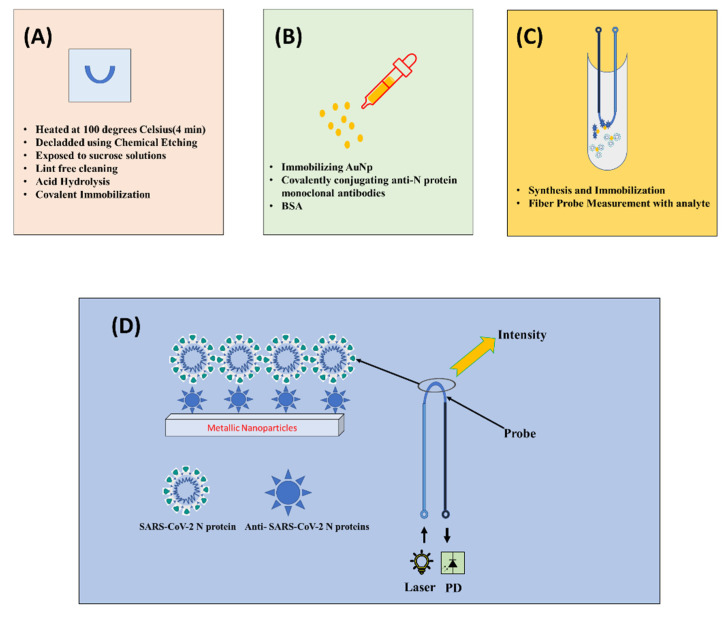
Block diagram of U-shaped fiber-optic sensing for SARS-CoV-2 detection general steps that are followed. (**A**) presents creation of U-shaped fiber; (**B**) represents functionality activation of U-shaped fiber probe; (**C**) represents metallic nanoparticles synthesis and immobilization and (**D**) presents the overall methodology for fiber-optic sensing for SARS-CoV-2 detection.

The utilization of fiber-optic sensors has found applications in chemical, civil, physical and health sectors. Since they are immune to electromagnetic interference, they offer higher sensitivity and detection limits. These characteristics make them advantageous over conventional schemes. One of the other advantages with optical fiber sensing is the provision of flexibility in configuration of fiber probes (D shaped, U shaped and tapered). Out of these possibilities, the U-shaped fiber probe configuration is the most efficient configuration for the localized plasmon response as it provides the quickest response compared to other probes [29]. The proposed U-shaped fiber probe method has excellent sensitivity in the range of parts per billion (ppb), with a response time ranging from 25 to 60 s [30,31]. The steps followed for the fiber-optic-based sensing is summarized step by step in sections below according to the recent work shown in [32,33]. Figure 4 also shows the schematic overview of steps involved.

### 4.1. Fabrication of U-Bent POF Probes

The plastic optical fiber consists of polymethylmethacrylate (PMMA) core and fluorinated polymer cladding with refractive indices (RI) of 1.49 and 1.41, respectively. Briefly, 25 cm long 200 and 500 µm POFs bent at the middle portion to get a U-shaped probe. The U-shaped fiber was heated at 100 degrees Celsius for 4 min to obtain a permanent deformation. The probe region was made by de-cladding the U-bent area using chemical etching achieved by incubating the probes in ethyl acetate for 2 min. Variations across probes were reduced by carefully selecting probes with a RI sensitivity of about 3.1. The POFs were exposed to sucrose solutions of various concentrations to test their RI sensitivity. The de-cladded portion was cleaned with lint-free cleaning wipes from Comtec for removal of particles or bubbles. The illustration of U-shaped fiber probe is shown in Figure 4, labeled as (A).

### 4.2. Metallic Nanoparticles Synthesis and Immobilization

The gold nanoparticles (AuNp) can be synthesized by citrate-mediated reduction of gold chloride. The biosensor matrix must first be produced by immobilizing gold nanoparticles on a U-bent fiber-optic probe by Satija et al. 2014 in [33,34], then covalently conjugating anti-N protein monoclonal antibodies. For this reason, high-affinity antibodies will be obtained. In addition, established methods such as ELISA will be used to determine the binding affinity and activity of the antibodies. The antibody-immobilized probes will next be treated with a solution of bovine serum albumin (BSA) to avoid nonspecific interactions. Following that, these biofunctionalized probes will be utilized to diagnose COVID-19 by introducing a saliva sample from the patient within 15 min. The use of gold nanoparticles further amplifies the interaction signal that eventually leads to a greater light interaction resulting in an increase in intensity. 

### 4.3. Functionality Activation of U-Bent POF Probes

The surface functionality of the fiber probe is obtained as explained in [32] where the U-bent probe was exposed to 0.5 M $H_{2}{\mathrm{SO}}_{4}$ for 1 min. By reducing the methyl ester groups by acid hydrolysis, this step is required to obtain carboxylic groups on the PMMA surface followed by the incubation in a 7% HMDA solution for half an hour at room temperature to create amine functional groups on the surface. They were then dipped in 1% Silane prepared in ethanol: acetic acid (10:4) solvent for 3–5 min in Argon ambient. Acetic acid helps in restricting the formation of multi-layers of silane that could lead to aggregation of gold nanoparticles on sensor surfaces. The probes were subjected to 2.5 percent glutaraldehyde for fifteen minutes for covalent immobilization. This step is shown in Figure 4 labeled as (B) and (C). 

### 4.4. Fiber-Optic Sensing and Signal Processing

It is well known that propagation of light via total internal reflection inside optical fiber is influenced by variation in the refractive index. Besides, the intrinsic evanescent wave in case of optical fiber is pinned to the core during removal of the cladding portion, which further facilitates interaction with the surrounding medium. A change in surroundings results in refractive index variation, which eventually leads to modulated light intensity. When relevant functionalities corresponding to the analyte are impregnated, the intensity fluctuation becomes more obvious. The exposed area is subsequently impregnated with appropriate functions. It is desirable to use noble metal nanoparticles that further immobilize with appropriate bio receptors/bio linkers and amplify the interaction signal, eventually leading to a greater metrics performance (intensity, absorbance, etc.). Antibodies/proteins might be used as bio receptors/bio linkers for SARS-CoV-2 in this suggested catheter-like probe. When the probe is exposed to the virus (analyte), it undergoes an intensity modulation. The behavior is explained in Figure 4 with the presence of metallic nanoparticles present on the upper layer. However, this can be a quite cost-consuming method so utilization of nanoparticles should be selected in a way that is economical and appropriate. 

The signal processing through the probe can be assessed through many methodologies, namely intensity modulation, wavelength division multiplexing, frequency multiplexing, etc. However, the intensity modulation is the quickest and most stable solution.

Considering detectors to be extremely accurate, if we consider the practicality of the schemes, the intensity-based interrogation approach might be considered the greatest fit because it requires the least amount of logistics; when the proposed configuration is complete, positive samples (infected) are tested in a controlled environment. Similarly, the same setup can also be made for samples without any infection. Accordingly, the Positive (SARS-CoV-2 infected) and negative (SARS-CoV-2 not infected) profiles can be correlated in order to set a threshold for positive cases identifications, thereby approximating the calibration procedure. 

The decision is expressed in the following way:

(1) $$I_{v}>I_{v}{}_{threshold}\;\Rightarrow \;\mathrm{SARS}-\mathrm{CoV}-2\;\mathrm{is}\;\mathrm{positive}$$

(2) $$I_{v}<I_{v}{}_{threshold}\;\Rightarrow \;\mathrm{SARS}-\mathrm{CoV}-2\;\mathrm{is}\;\mathrm{negative}$$

where $I_{v}$ represents the attained intensity of the sample or swab under test while $I_{v}{}_{threshold}$ shows the threshold intensity.

The proposed probing can be used in both oropharyngeal swab and nasopharyngeal swab methods. When the probe encounters the swab, modulated intensities are triggered. If the attained intensity $I_{v}$ abides by Equation (1) then the sample is COVID-19 positive while if $I_{v}$ is below $I_{v}{}_{threshold}$ than the sample is declared COVID-19 negative.

Similarly, the viral load is included in the proposed system based on the modulated response and calibration from the NP standard. The handheld unit can be equipped with wireless capability that can be incorporated into this method. The two approaches are quite viable/adoptable, depending on the receptor for functionalizing the probes. The modulated responses acquired with respect to the swabs are used to establish the cases’ positivity. The optimization of the specimen with several species of viruses belonging to the SARS-CoV-2 family will be carried out before it is used for direct sample analysis. If the modulated response is above the threshold, the diagnosis is complete, enabling avoidance of false-positive results at the same time. This step is shown as Figure 4 and labeled as step (D).

## 5. Proposed Experimental Testbed

**Figure 5:**
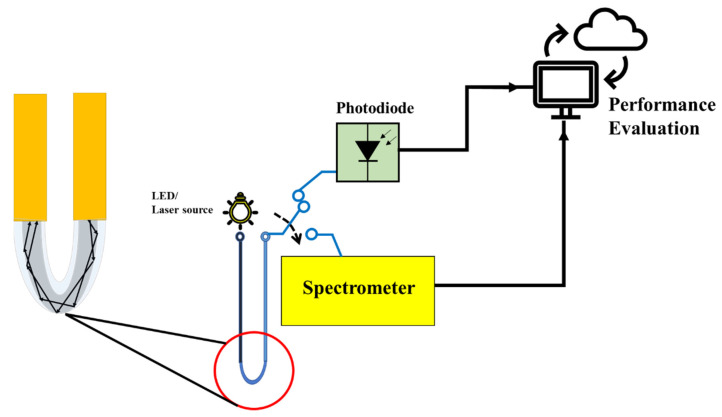
Schematic diagram of U-shaped optical fiber probe connected on one side with LED laser source and other probe with the photodiode or spectrometer. The performance is then evaluated.

The experimental testbed is designed to perform the tests as shown in Figure 5. The variations in power sensors is a severe concern due to drift in the utilized U-shaped optical fiber probe. However, in order to reduce this effect, a plastic over fiber is employed. In this study, two POF with diameters 200 and 500 µm were used one by one. The plastic optical fiber consisted of polymethylmethacrylate (PMMA) core and fluorinated polymer cladding with refractive indices of 1.49 and 1.41, respectively. Briefly, 25 cm long 200 and 500 µm POFs bent at the middle portion to get a U-shaped probe.

A Thorlabs M530F2 @ 200 mA laser source was utilized [34]. The electrical power was 3100 mW while the typical output power is 9.6 mW for 500 and 3.2 mW for 200 µm fiber diameter. 

The probe is exposed to the sample under test that can be an oropharyngeal or nasopharyngeal swab. The other side of probe is connected to Spectrometer or Photodiode that can evaluate the performance metric. The decision of COVID-19 positivity is made according to the Equations (1) and (2) when intensity is greater than the threshold intensity. The data were calculated with a 1 ms integration time, and the average of 1000 samples were provided. The parameters utilized in this work are summarized in Table 2.

## 6. Theoretical Understanding behind Wavelength-Bending Radius and Wavelength Refractive Index Relationship

### 6.1. The Wavelength–Bend Radius Relation

When SMF experience bending, Renner et al [35]. proposed that the transverse field distribution φ(x,y) is expressed as:

(3) $$\nabla_{t}^{2}\;\varphi \left(x,y\right)+\left[k^{2}n_{eff}^{2}\left(x,y\right)-\beta^{2}\right]\varphi \left(x,y\right)=0\;$$

where $k=\frac{2\pi }{\lambda }$, and β represents the propagation constant and λ represents the wavelength of the basic leakage mode. $n_{2}$ and $n_{3}$ are the refractive indices of the cladding and the surface coating. The radii of the core and cladding are represented by a and b, respectively. For x≥a; the effective refractive index in the bent fiber is given as:

(4) $$n_{eff}^{2}\left(x,y\right)=n^{2}\left(x,y\right)\left(1+\frac{2x}{R}\right)$$

where $n^{2}\left(x,y\right)$ represents the refractive index of the unbent optical fiber. The effective refractive index $n_{eff}\left(x,y\right)$ changes according to the diameter (x) and the bend radius (R) of the optical fiber. Since optical fibers of two diameters were used in this study, Equation (4) becomes:

(5) $$\lambda \times {\left(\Delta \lambda \right)}^{-\frac{1}{2}}={\left[\frac{4n_{eff}^{2}}{3}\;\sqrt{\frac{2b^{3}}{R}}\;\right]}^{\frac{1}{2}};R\ll R_{c}\;$$

when $R\ll R_{c}$, the ratio of the wavelength position can be derived from the bend radius (*R*), the radius of the cladding (*b*), and the effective refractive index ($n_{eff}$). According to the previous wavelength-related equations, the wavelength shifts are primarily affected by three parameters—the diameter of the optical fiber, the bend radius and the effective refractive index.

### 6.2. The Wavelength–Refractive Index Relation

The sensitivity of the refractive index to wavelength is discussed in this section. A leakage mode arises in the bending zone of an optical fiber with a bend radius small enough that when light enters the bending region, total reflection is no longer maintained at the interface between the core and cladding layers, according to Lu et al. [36] and Zhang et al. [37]. As a result, certain light rays from the core layer can leak or be released to the cladding layer, reflecting at the interface between the external medium and the cladding layer, generating a whispering gallery mode (WGM). After then, the light beams would be linked back to the core layer. Due to the two separate transmission paths, interference is created when light beams couple via the WGM from the cladding layer to the core layer. As a result, a unique WGM wavelength spectrum is created, with various peaks and troughs visible.

The interference spectrum light source intensity of the WGM can be represented by Equation (6) [38].

(6) $$I=I_{co}+I_{w}+2\sqrt{I_{co}+I_{w}}\;cos\left(\phi \right)\;$$

where $I_{co}$ and $I_{w}$ are the light intensities in the fiber’s core mode and WGM, respectively, and ϕ is the phase difference between the core mode and the WGM. It is well understood that increasing the refractive index changes the effective refractive index difference between the core and cladding, as well as the position of the wavelength at the point of loss. Based on the refractive index fluctuations in the external environment, the wavelength sensitivity can be stated as:

(7) $$\frac{d\lambda_{D}}{dn_{ext}}=-\frac{\lambda_{D}}{\Delta n_{eff}}\frac{\partial_{n_{eff}}^{cl,m}}{\partial n_{ext}}/\left[1-\frac{\lambda_{D}}{\Delta n_{eff}}\left(\frac{\partial_{n_{eff}}^{co}}{\partial \lambda }-\frac{\partial_{n_{eff}}^{cl,m}}{\partial \lambda }\right)\right]\;$$

The equations presented above linking the refractive index and sensitivity reveal that when the external refractive index changes, the effective refractive indices of the core and cladding modes should change, causing wavelength shifts. The core of a typical single-mode optical fiber has a higher effective refractive index than its cladding, and its effective refractive index equation is $n_{eff}^{co}-n_{eff}^{cl,m}>0$.

Furthermore, when $\frac{\lambda_{D}}{\Delta n_{eff}}\left(\frac{\partial_{n_{eff}}^{co}}{\partial \lambda }-\frac{\partial_{n_{eff}}^{cl,m}}{\partial \lambda }\right)<$1, the wavelength sensitivity to refractive index fluctuations is negative, implying that any increase in the refractive index decreases the wavelength.

Based on this explanation in this section, in case of a specimen with infection, it is observed that for a smaller diameter of 200 µm PoF, the intensity curve is much stronger compared to the case when 500 µm diameter PoF is utilized. This shows that the correlation of the specimen with a smaller-diameter probe is better as the intensity is higher due to better exposure and higher total internal reflection. Similarly, the bending in terms of U-shaped fiber is better compared to D- or T-shaped probe fiber. 

## 7. Results

**Figure 6:**
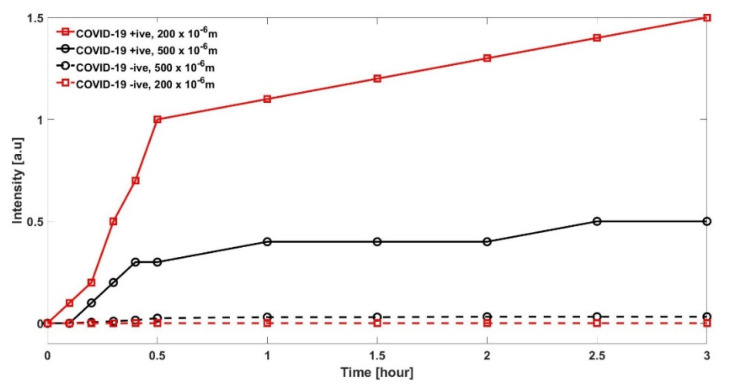
Comparison of two POFs in terms of intensity with diameters 200 and 500 µm are compared with specimen with a COVID-19 positive and negative result.

The experimental results are evaluated for a specimen from a person with and without COVID-19. Firstly, the results in Figure 6 reports the intensity measured for two different diameters of PoFs under test. In case of specimen with infection, it is observed that, for a smaller diameter of 200 µm PoF, the intensity curve is much stronger as compared to the case when 500 µm diameter PoF is utilized. This is in line with the explanation made in Section 6. The only reason for comparing two diameters PoF is to justify that smaller diameter is useful. 

**Figure 7:**
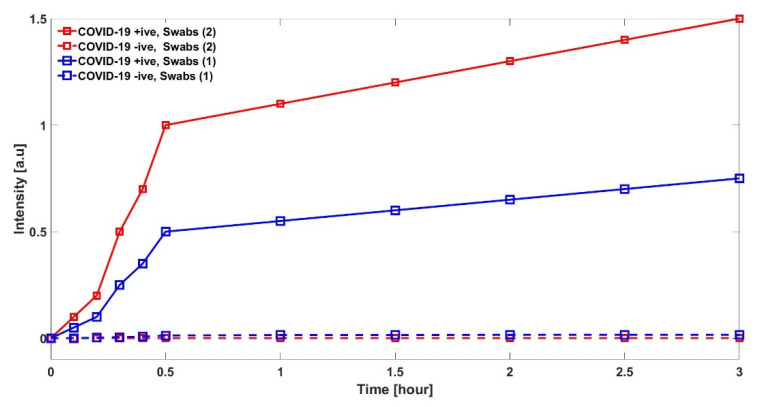
Comparison of two POFs in terms of intensity with diameter 200 µm, compared with specimen taken from oropharyngeal or/and nasopharyngeal.

In Figure 7, we have compared the results when swabs are taken from both oropharyngeal or nasopharyngeal (labeled as Swab (2)) and compare them with a single swab, either oropharyngeal or nasopharyngeal (labeled as Swab (1)). This confirms that the proposed method works in both orientations; however, the trends intensities are different. It should be noted that detection time window is similar for both tests, i.e., within the first 15 min. 

**Figure 8:**
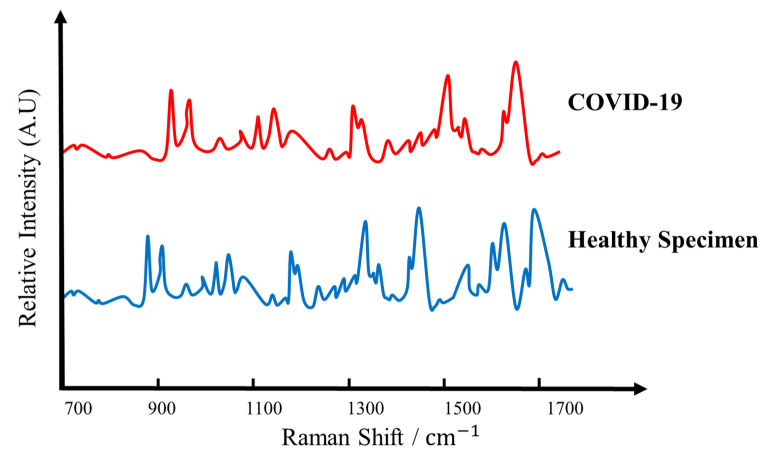
Comparison of 200 µm POF with COVID-19 specimen and healthy specimen evaluated in terms of Raman Peaks.

In Figure 8, a spectrometer measures the vibrations at the interatomic level that are produced once a laser diode sheds light on the specimen. Each virus has its own signature vibrations, which act as a sort of optical fingerprint that can distinguish the coronavirus from other specimens. When a laser diode via a U-shaped probe sheds the light on the specimen under test, COVID-19 infected specimen creates a distinctive optical fingerprint, or “Raman peaks” as shown in Figure 8. This leads to the conclusion that this setup will also be able to detect other variants of COVID-19 such as Omicron in a similar fashion.

## 8. Technique Validation

**Figure 9:**
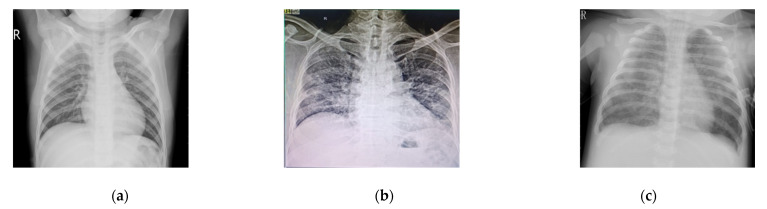
X-ray of (**a**) normal patient with no COVID-19 symptoms for pelvis surgery with negative PCR test; (**b**) is a patient with COVID-19 symptoms, positive PCR test on day 1 and 3; (**c**) is a pneumonia patient with negative PCR test on day 1, 3 and 5.

In order to validate the proposed technique, it is important to verify that the proposed technique detects SARS-CoV-2 and not something random. In this research, the sample consisted of COVID-19 patients which presented to the hospital with respiratory symptoms. Amongst those, the ones in which the virus progressed to pneumonia leading to radiographic changes detected in chest X-rays/CBCT scans were selected for the study. For this, we have considered three specimens and their X-rays are shown. In Figure 9a, a normal patient X-ray is shown of a patient who came to the hospital for a pelvic surgery and was PCR tested, which provided a negative result 12 h before this X-ray. The patient had no COVID-19 symptoms. Figure 9b presents the X-ray of a patient who had a COVID-19 symptoms with breathing difficulties. The PCR was performed on day 1 and day 3 and provided a positive result. The radiologist also confirmed that X-ray suggested it was a COVID-19 picture. Similarly, Figure 9c shows an X-ray of a pneumonia patient; the PCR test was negative on day 1, 3 and 5 of observation confirming that it was not a COVID-19 case. The radiologist and professor of medicine suggested that it was a pneumonia case.

**Figure 10:**
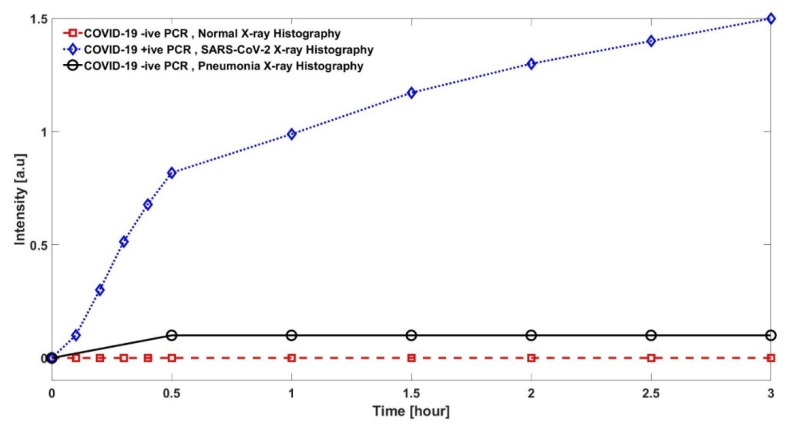
Comparison of Normal, COVID-19 and Pneumonia patients in terms of intensity with diameters 200 µm POF are compared.

In order to see the comparison of specimens under test in terms of intensity, oral swabs were taken from patients with 200 microns diameter of PoF. Figure 10 reports the intensity of the specimens, while we already know their histopathology. The results clarify that the proposed approach has a different intensity for different specimens based on SARS-CoV-2, Pneumonia and Normal patients. SARS-CoV-2 has a very clean and high intensity, which is higher compared to Pneumonia patients with a very clear difference, while a normal patient has intensity almost equal to zero. As far as the question is concerned of whether it detects other viruses, this is a broad question and at this moment the only concern that we have is to offer a fast and reliable detection system for the pandemic. In future, the detection of other COVID-19 variants such as the Omicron variant using the same technique is envisaged. 

## 9. Statistical Analysis

**Figure 11:**
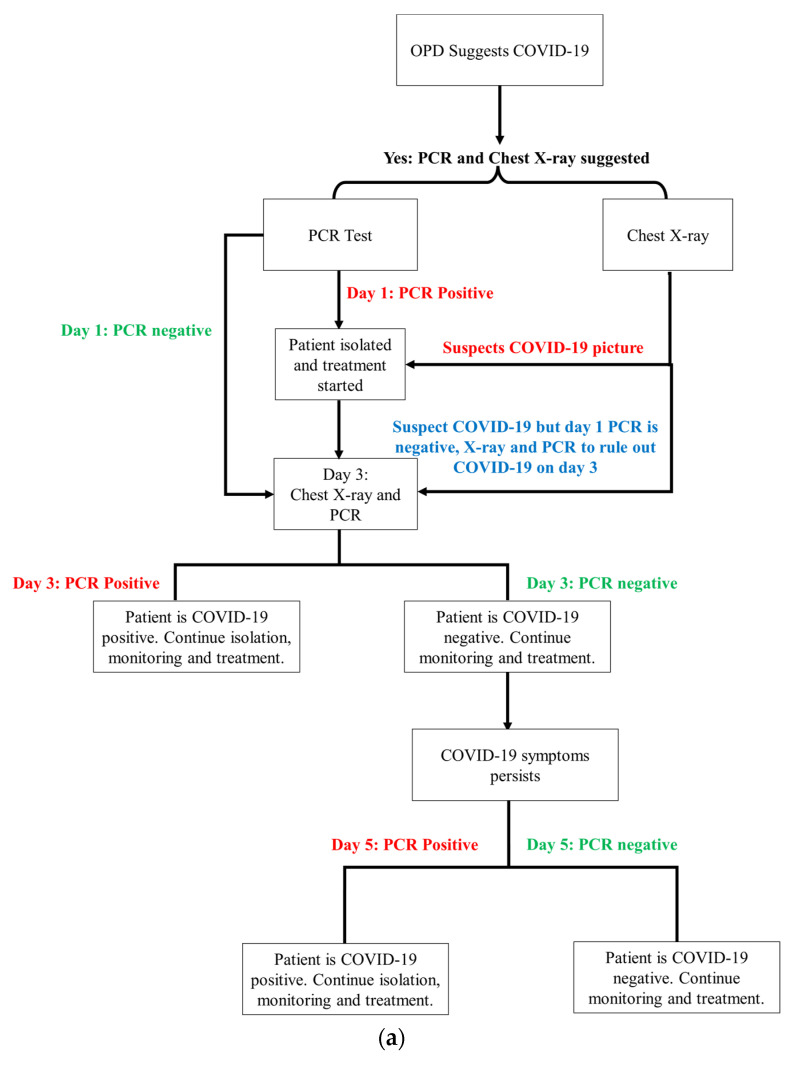

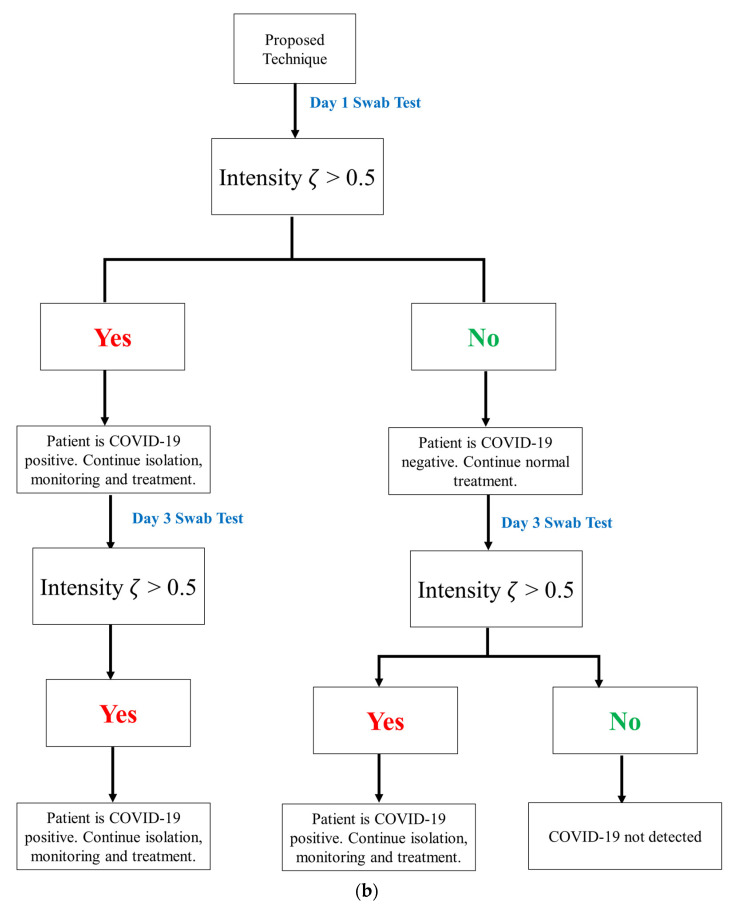
(**a**) Process flow of the conventional COVID-19 detection followed in the hospital under observation; (**b**) process flow of the proposed early detection of COVID-19.

In order to show the statistical analysis of the results obtained, it is important to understand the cycle or map that is followed for COVID-19 detection in the hospital compared to the cycle of actions required in proposed detection. The scheme in Figure 11a summarizes the process flow of the COVID-19 detection in the conventional method compared to Figure 11b that includes the process flow for early COVID-19 detection in the proposed technique. If the intensity is below the set threshold, then it is a negative COVID-19 specimen (patient). Still, to be on safe side, and, as this is a research phase, it is advised to continue monitoring and test the patient again on day 3 (two days after first test) to check if there is any change in intensity and if the patient tests positive. The results shown in Table 3 validate that there has never been a false detection for the samples collected on day 1; however, there is the process flow available if a patient tests COVID-19 positive on the third day. 

In this research, the sample consisted of COVID-19 patients which presented to the hospital with respiratory symptoms. Amongst those, the ones in which the virus progressed to pneumonia leading to radiographic changes detected in chest X-rays/CBCT scans were selected for the study.

Table 3 has been added, which shows the chest X-ray prognosis, PCR tests on day 1, 3 and 5 and proposed technique findings. The results obtained with the conventional methodology result in false and wrong detection. This can occur at three stages, which are as follows:

- GP stage where an Outpatient Department (OPD) based on symptoms refers for further PCR and X-ray chest to rule out the COVID-19.
- PCR test is negative on day 1 (the day of report) while the symptoms are clearly providing a COVID-19 picture and the X-ray is also suggesting COVID-19.
- PCR test has a negative result on day 3 again, while COVID-19 is suggested from the chest X-ray.

The statistical data obtained from the data are given in Table 4. The table summarizes that there is 32 and 16% false detection on day 1 with PCR test and chest X-ray synopsis, respectively, while this number reduces to 8% of false detection with a PCR test on day 3 and drops to 0% on PCR test repetition on day 5. The test environment is the same and the method of testing is similar too. It is important to note that the proposed technique results in 0% false detection on day 1, which means that this is a fast and accurate detection method. 

## 10. Future Work and Direction

**Figure 12:**
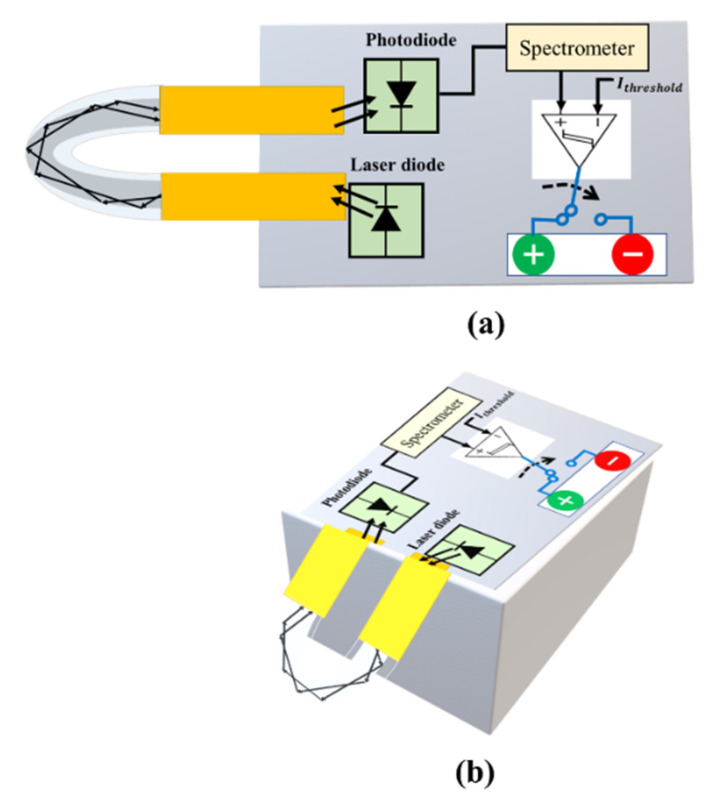
The proposed prototype will have a following shape. The U-shaped optical fiber probe connected to the laser and photodiode while green light shows a COVID positive, and COVID negative is reported by red light. (**a**) Shows front view while (**b**) shows lateral view of the prototype.

Finally, the proposed optical technology can detect COVID-19 and assist medical personnel in real time, enhancing efficiency and accuracy. As shown in Figure 12, the proposed prototype will produce a qualitative analysis either in terms of intensity or Raman scattering, creating the possibility of broad testing and feasibility in either of the modes used in the prototype. To put it another way, a patient’s confirmation, whether positive or negative, can be determined. There is every possibility of measuring the trace of SARS-CoV-2 with a little bit of upgradation/tweaking during calibration prior to real-time testing. This will aid in determining the infection level in the patient’s body. Alternatively, the prototype can be used to assess distinct traces of SARS-CoV-2 spiked samples, resulting in varied intensity levels. The intensity levels are recorded by the photodiode connected to the other side of the probe. 

Taking these magnitudes of modulated intensities into account, the prototype can also be used for quantitative estimation/assessment, as shown in Figure 9. Both plans can benefit from this. Apart from that, the scheme’s portability and small size allow it to be readily integrated with wireless capabilities via Internet of Things (IoT) applications such as smartphones, wireless information handling, etc. As a result, the test results can be simply stored away in cloud storage for later e-health care administration. Although the proposed approach may be a realistic technique for assessing COVID-19 cases quickly, real-world testing of the probe would have been more conclusive. It is certain that the proposed system for sensing real samples will be adopted and implemented in the near future. Financial assistance and time will be required to create the prototype-based quick, portable COVID-19 diagnostic tool. The financial assistance will make it easier to complete the project. Mass fiber-optic production, point-of-care (PoC) devices and probes and cartridges production is required, and this cannot be achieved without industrial and financial assistance. To handle viral particles and clinical samples, as well as to continue, a laboratory setup might be used to validate the assay and PoC device for SARS-CoV-2 detection in saliva samples. Industrial assistance is required in the following:

(i) To build process flow lines for the above-mentioned cartridges.
(ii) To create user-friendly gadgets, probes, and other tools.
(iii) For proof-of-concept devices, a third-party validation of the device is required.
(iv) To build distribution routes for the greatest reach.

## 11. Conclusions

In this article, a novel plastic optical fiber sensor U-shaped probe sensing method is envisaged for detection of SARS-CoV-2, commonly known as COVID-19 virus, that also has the capability to detect new variants such as Omicron. This specific fiber-optic probe for SARS-CoV-2 can provide quick diagnosis via insertion into the oropharyngeal and nasopharyngeal tract thanks to the synergistic integration of improved photonics and the addition of appropriate biomarkers. The sensing approach based on intensity modulation appears to be very cost effective with minimum logistics. This one-of-a-kind adaption is extremely simple to operate and does not necessitate the usage of trained personnel. The envisaged prototype version can be simply connected to a national health services system for reporting, self-analysis and limiting the COVID breakout, eliminating the inconveniences that patients have while visiting established/registered clinics/labs/hospitals. The prototype may be adjusted to perform quantitative assessments via controlled standardization versus intensity magnitudes against spiking SARS-CoV-2 infection, in addition to qualitative assessments. The results presented in terms of relative intensity and intensity suggest that plastic over fiber of 200 microns in diameter has better detection accuracy compared to a higher diameter. It was also shown that the detection of other variants of COVID-19 will be applicable. 

## Figures and Tables

**Table 1:** Diagnostic techniques to detect SARS-CoV-2.

No.	Type	Details	Efficiency (η)	Pros	Cons
1	IgM / IgG Rapid Test	Immunoassays are tests that identify specific antibodies in the patient’s blood. A lateral flow immunoassay has been developed that can detect IgM and IgG in human blood just in 15 min	COVID-19 IgM / IgG Rapid Test Sensitivity is 88.66% [23]	Employing a synthetic peptide as an antigen improves the immunoassay’s stability and reproducibility and is theoretically more specific than using a virus as an antigen [24].	Typically, the immunoassay yields only qualitative results.
2	Polymerase Chain Reaction (PCR) Test	The testing technique involves the following steps: I specimen collection; (ii) clinical specimen packaging (storage) and transportation; (iii) (good) communication with the laboratory and giving the necessary information; (iv) laboratory testing; (v) reporting the results [25].	The time it takes to get the results can be as long as two or three days [25]. Only 66 to 80% of it is sensitive [23].	The rRT-PCR technique necessitates expensive laboratory equipment, which is frequently found in a central laboratory (biosafety level 2 or higher). As a result, the outcome is extremely dependable.	Commercial PCR-based procedures are costly and reliant on technical knowledge, and the presence of viral RNA or DNA does not always indicate acute illness.
3	A disease diagnosis model based on radio and clinical characteristics [26].	Pneumonia patients with and without COVID-19 can be identified only based on CT imaging and clinical symptoms. These models will be critical for early and easy-to-access diagnosis, especially when RT-PCT kits or experimental platforms to screen for COVID-19 infection are in short supply [27].	In primary and cohort validation, the area under the curve was 0.986 (95 percent confidence interval 0.966 1.000) and 0.936 (95 percent confidence interval 0.866 1.000), respectively [27].	The clinical and radiological semantic models performed better in terms of diagnostic accuracy and yielded greater net benefits.	According to the study, 18 radiological and 17 clinical characteristics were determined to be relevant in forming COVID-19 infection predictions.
4	(Loop-mediated isothermal amplification) LAMP assay	A set of four specially designed primers and a DNA polymerase with strand displacement activity are used in this procedure. Instead of heat denaturation, LAMP generates a single-stranded template using strand displacement polymerase [27]	At a steady temperature of 65 °C, LAMP can manufacture up to 109 copies of target DNA in less than an hour. Detection sensitivity is greater than 95%.	LAMP has a good sensitivity and is simple to execute, but it also has the potential to run at a fixed temperature, which reduces the phenomenon effects of a thermocycler while also reducing the amount of energy used.	The LAMP technique’s clinical usefulness for SARS-CoV-2 has yet to be investigated.
5	Luminescent Immunoassay	Luminescent immunoassays are approaches for lowering the detection limits of antibody-based reagents. Chemiluminescence and fluorescence are commonly used [28]	IgG was found in 71.4 percent of all sera (197/276), which is 192 percent greater than IgM (57.2 percent, 158/276). The detected rate was increased to 81.5 percent (225/276) when the two 193 antibodies were combined. In SARS, different sensitivities of the 194 IgG and IgM detection techniques were recorded.	Diazyme Laboratories Inc. has revealed details of two new fully automated SARS-CoV-2 serological assays that can be performed on the Diazyme DZ-lite 3000 Plus chemiluminescence analyzer.	It is currently approved for usage in the United States, China, and Brazil
6	Biosensing Method	Biosensor tests rely on optical, electrical, enzymatic and other techniques to translate the unique activity of biomolecules into a quantitative output [28].	Within 10 min, the surface plasmon resonance (SPR) chip detected anti-SCVme antibodies at a lower limit of detection of 200 ng/mL.	PathSensors Inc. recently developed a CANARY biosensor to detect the new SARS coronavirus. This approach makes use of a cell-based immunosensor that combines viral collection with signal amplification to produce a result in 35 min.	In May 2021, the biosensor will be available for research purposes.

**Table 2:** Testbed parameters.

Parameters	Values
Laser	Wavelength = 530 nm Electrical Power= 3100 mW $\mathrm{Output}\;\mathrm{Power}=\{\begin{array}{c} 3.2\;\mathrm{mW}\;@200\;µm\;\;\\ 9.6\;\mathrm{mW}\;@\;500\;µm \end{array}$
Optical Fiber	Type = POF Diameter = 200 and 500 µm
Photodiode	Responsivity = 0.34 A/W Bandwidth =2 GHz

**Table 3:** Collected data for 25 samples with different stages OPD symptoms history, PCR results for day 1 (day of observation), 3 and 5 with our proposed technique results.

Sample	OPD Symptoms Suspect	PCR Test Day 1	X-Ray Suspect	PCR Test Day 3	PCR Test Day 5	Proposed Technique Suspect Test Day 1	Proposed Technique Suspect Test Day 3
1	COVID-19	Positive	COVID-19	Positive	Positive	COVID-19	COVID-19
2	COVID-19	Negative	COVID-19	Positive	Positive	COVID-19	COVID-19
3	COVID-19	Negative	COVID-19	Positive	Positive	COVID-19	COVID-19
4	COVID-19	Positive	COVID-19	Positive	Positive	COVID-19	COVID-19
5	COVID-19	Positive	COVID-19	Positive	Positive	COVID-19	COVID-19
6	COVID-19	Negative	Pneumonia	Positive	Positive	COVID-19	COVID-19
7	COVID-19	Negative	Pneumonia	Negative	Negative	No	No
8	COVID-19	Positive	COVID-19	Positive	Positive	COVID-19	COVID-19
9	COVID-19	Negative	Pneumonia	Negative	Negative	No	No
10	COVID-19	Positive	COVID-19	Positive	Positive	COVID-19	COVID-19
11	COVID-19	Negative	Pertussis	Negative	Negative	No	No
12	COVID-19	Negative	Pneumonia	Negative	Negative	No	No
13	COVID-19	Negative	COVID-19	Positive	Positive	COVID-19	COVID-19
14	COVID-19	Negative	COVID-19	Negative	Negative	No	No
15	COVID-19	Positive	COVID-19	Positive	Positive	COVID-19	COVID-19
16	COVID-19	Positive	COVID-19	Positive	Positive	COVID-19	COVID-19
17	COVID-19	Negative	Pneumonia	Negative	Positive	COVID-19	COVID-19
18	COVID-19	Positive	COVID-19	Positive	Positive	COVID-19	COVID-19
19	COVID-19	Negative	COVID-19	Positive	Positive	COVID-19	COVID-19
20	COVID-19	Negative	COVID-19	Positive	Positive	COVID-19	COVID-19
21	COVID-19	Negative	Pneumonia	Positive	Positive	COVID-19	COVID-19
22	COVID-19	Positive	COVID-19	Positive	Positive	COVID-19	COVID-19
23	COVID-19	Positive	COVID-19	Positive	Positive	COVID-19	COVID-19
24	COVID-19	Negative	COVID-19	Positive	Positive	COVID-19	COVID-19
25	COVID-19	Negative	COVID-19	Negative	Positive	COVID-19	COVID-19

**Table 4:** Statistical analysis of false detection with conventional and proposed techniques.

Specification	Value
Total Number of Samples	25
PCR Test Day 1 Missed Detection	8
X-rays Synopsis Missed Detection	4
PCR Test Day 3 Missed Detection	2
PCR Test Day 5 Missed Detection	0
Proposed Technique Test Day 1 Missed Detection	0
Proposed Technique Test Day 3 Missed Detection	0

## Data Availability

Not applicable.
